# Directional cerebrospinal fluid movement between brain ventricles in larval zebrafish

**DOI:** 10.1186/s12987-016-0036-z

**Published:** 2016-06-21

**Authors:** Ryann M. Fame, Jessica T. Chang, Alex Hong, Nicole A. Aponte-Santiago, Hazel Sive

**Affiliations:** Whitehead Institute for Biomedical Research, Nine Cambridge Center, Cambridge, MA 02142 USA; Massachusetts Institute of Technology, 77 Massachusetts Avenue, Cambridge, MA 02139-4307 USA

**Keywords:** Cerebrospinal fluid, Brain ventricular system, Fluid dynamics, Zebrafish

## Abstract

**Background:**

Cerebrospinal fluid (CSF) contained within the brain ventricles contacts neuroepithelial progenitor cells during brain development. Dynamic properties of CSF movement may limit locally produced factors to specific regions of the developing brain. However, there is no study of in vivo CSF dynamics between ventricles in the embryonic brain. We address CSF movement using the zebrafish larva, during the major period of developmental neurogenesis.

**Methods:**

CSF movement was monitored at two stages of zebrafish development: early larva [pharyngula stage; 27–30 h post-fertilization (hpf)] and late larva (hatching period; 51–54 hpf) using photoactivatable Kaede protein to calculate average maximum CSF velocity between ventricles. Potential roles for heartbeat in early CSF movement were investigated using *tnnt2a* mutant fish (*tnnt2a*^−/−^) and chemical [2,3 butanedione monoxime (BDM)] treatment. Cilia motility was monitored at these stages using the Tg(*βact:Arl13b*–GFP) transgenic fish line.

**Results:**

In wild-type early larva there is net CSF movement from the telencephalon to the combined diencephalic/mesencephalic superventricle. This movement directionality reverses at late larval stage. CSF moves directionally from diencephalic to rhombencephalic ventricles at both stages examined, with minimal movement from rhombencephalon to diencephalon. Directional movement is partially dependent on heartbeat, as indicated in assays of *tnnt2a*^−/−^ fish and after BDM treatment. Brain cilia are immotile at the early larval stage.

**Conclusion:**

These data demonstrate directional movement of the embryonic CSF in the zebrafish model during the major period of developmental neurogenesis. A key conclusion is that CSF moves preferentially from the diencephalic into the rhombencephalic ventricle. In addition, the direction of CSF movement between telencephalic and diencephalic ventricles reverses between the early and late larval stages. CSF movement is partially dependent on heartbeat. At early larval stage, the absence of motile cilia indicates that cilia likely do not direct CSF movement. These data suggest that CSF components may be compartmentalized and could contribute to specialization of the early brain. In addition, CSF movement may also provide directional mechanical signaling.

**Electronic supplementary material:**

The online version of this article (doi:10.1186/s12987-016-0036-z) contains supplementary material, which is available to authorized users.

## Background

Cerebrospinal fluid (CSF) is contained in the brain ventricles (chambers derived from the brain lumen) and contacts neuroepithelial progenitor cells during brain development and in adulthood. The CSF contains a unique composition of proteins (CSF proteome) [1–6], small molecules [5, 7] and lipid particles [8] for which limited studies have demonstrated function during brain development [1–5, 7–9]. Since the brain lumen forms before the choroid plexus (CP), ventricles exist with no requirement for a CP to be associated with each ventricle. In some cases early pre-CP fluid is called ependymal fluid [10]. The cohort of proteins in zebrafish CSF is largely overlapping with early amniote CSF prior to CP development [7] and this conservation has not been investigated at later stages. Once the CPs have become functional they secrete most of the ventricular fluid, although there is clear data that fluid continues to be produced from ependymal cells even after CP differentiation is complete [11–14]. The ventricular fluid is thus always a mix from different sources and the term CSF is most general and appropriate to refer to the ventricular fluid at all stages.

Restricted or directional CSF movement, either during production and drainage or coupled to heartbeat [15–19], could limit locally-produced factors to specific regions of the developing brain. Studies in *Xenopus* have investigated local intraventricular CSF dynamics in the developing brain [19, 20], but there has been no direct study of in vivo CSF movement dynamics between ventricles. Recent studies have suggested that cilia on neuroepithelial cells may detect a mechanical signal to regulate neuronal progenitor differentiation [21]. The nature of this signal is unknown but could be CSF movement. CSF fluid dynamics have been examined in zebrafish after CP formation [22], but have not been studied earlier in development during the major period of developmental neurogenesis. To address these deficits, we investigate CSF movement in the accessible larval zebrafish brain, prior to CP formation. Our data demonstrate directional movement of CSF, which may function during brain development.

## Methods

### Fish lines and maintenance

*Danio rerio* fish were raised and bred according to standard methods [23]. Embryos were kept at 28.5 °C and staged according to Kimmel et al. [24]. Times of development are expressed as hours post-fertilization (hpf) or days post-fertilization (dpf). Analyses were performed at early larva (pharyngula stage; 27–30 hpf) stage, late larva (hatching period; 51–54 hpf) stage, and some at 5 dpf. Lines used were: AB; Tg(*βact:Arl13b*–GFP) transgenic line [25]; *troponin T type 2a* (cardiac) mutants *tnnt2a*^−/−^ (R14GBT0031/silent heart; obtained from The Zebrafish International Resource Center (ZIRC; Eugene, USA)) [26]. Larvae were incubated in 3 μg/mL 1-phenyl-2-thiourea (PTU) starting between 22 and 24 hpf to inhibit maturation of melanocytes and allow for clear visualization. All experimental procedures on live animals and embryos were reviewed and approved by the Institutional Animal Care and Use Committee of the Massachusetts Institute of Technology (Protocol# 0414-026-17) and were carried out in accordance with the recommendations in the National Institutes of Health (NIH) Guide for the Care and Use of Laboratory Animals.

### Multiview SPIM brain ventricle imaging

Larvae were anesthetized in 0.1 mg/mL (0.38 mM) Tricaine (Sigma, St. Louis, USA) dissolved in embryo medium [23] prior to injection and imaging. At high doses, Tricaine can induce death or stop the heart [27–29], but these effects have not been shown at the concentration used here. Direct effects of Tricaine on CSF movement have not been investigated.

The rhombencephalic ventricle was microinjected with 2 nL of 2000 kDa dextran conjugated to fluorescein (ThermoFisher/Invitrogen Molecular Probes, Waltham, USA; D7137) diluted to 25 mg/mL in sterile ddH_2_O. Embryos were embedded in 1 % low-melting point agarose in a glass capillary, and selective plane illumination microscopy (SPIM) was performed using a Zeiss LightsheetZ.1 microscope within 60 min of injection. Six multi-view images were acquired (60**°** apart), and were processed using ZEN software (Zeiss, Oberkochen, Germany), FIJI [30, 31], and the Multiview reconstruction plugin [32, 33]. Images were analyzed using Imaris software (Bitplane, Belfast, UK) to visualize 3-D images and to generate volume measurements for ventricles at each age. The image threshold was set so that both the ventricle outline and the spinal canal were detected. For rhombencephalic ventricular volume measurements, the same threshold as above was used, but only the rhombencephalon was included in the region of interest. Injection does not affect development, survival or ventricle size over the time period studied ([34] and Additional file 1: Fig. S1).

### Tissue histology and hematoxylin and eosin staining

Larvae were anesthetized as described above and fixed overnight in aqueous Bouin’s fixative (RICCA Chemical, Pokomoke City, USA). Larvae were washed in 70 % EtOH until supernatant was clear (at least six washes of 1 h each at room temperature). Larvae were oriented in Richard Allan Scientific HistoGel (ThermoFisher, Waltham, USA). The HistoGel discs containing oriented larvae were fixed for 1 h in Bouin’s fixative, and subsequently washed in 70 % EtOH until supernatant was clear. Samples were infiltrated with paraffin using a Tissue-Tek VIP5 Vacuum Infiltration Processor (Sakura Finetek, Torrance, USA; model 5215). Sections were cut at 4–5 μm and slides were stained with Harris acidified hematoxylin (3 min) and eosin Y (10 s) using the “standard” H&E protocol [35] on a Shandon Varistain Gemini Slide Stainer (ThermoFisher, Waltham, USA). Slides were imaged using an Axioplan 2 (Zeiss, Oberkochen, Germany) equipped with a Q-Capture MicroPublisher 5.0 RTV camera/software (QImaging Scientific, Surrey, Canada) using 10× and 40× air objectives and 63× and 100× oil-immersion objectives.

### Live time-lapse confocal imaging

**Fig. 1:**
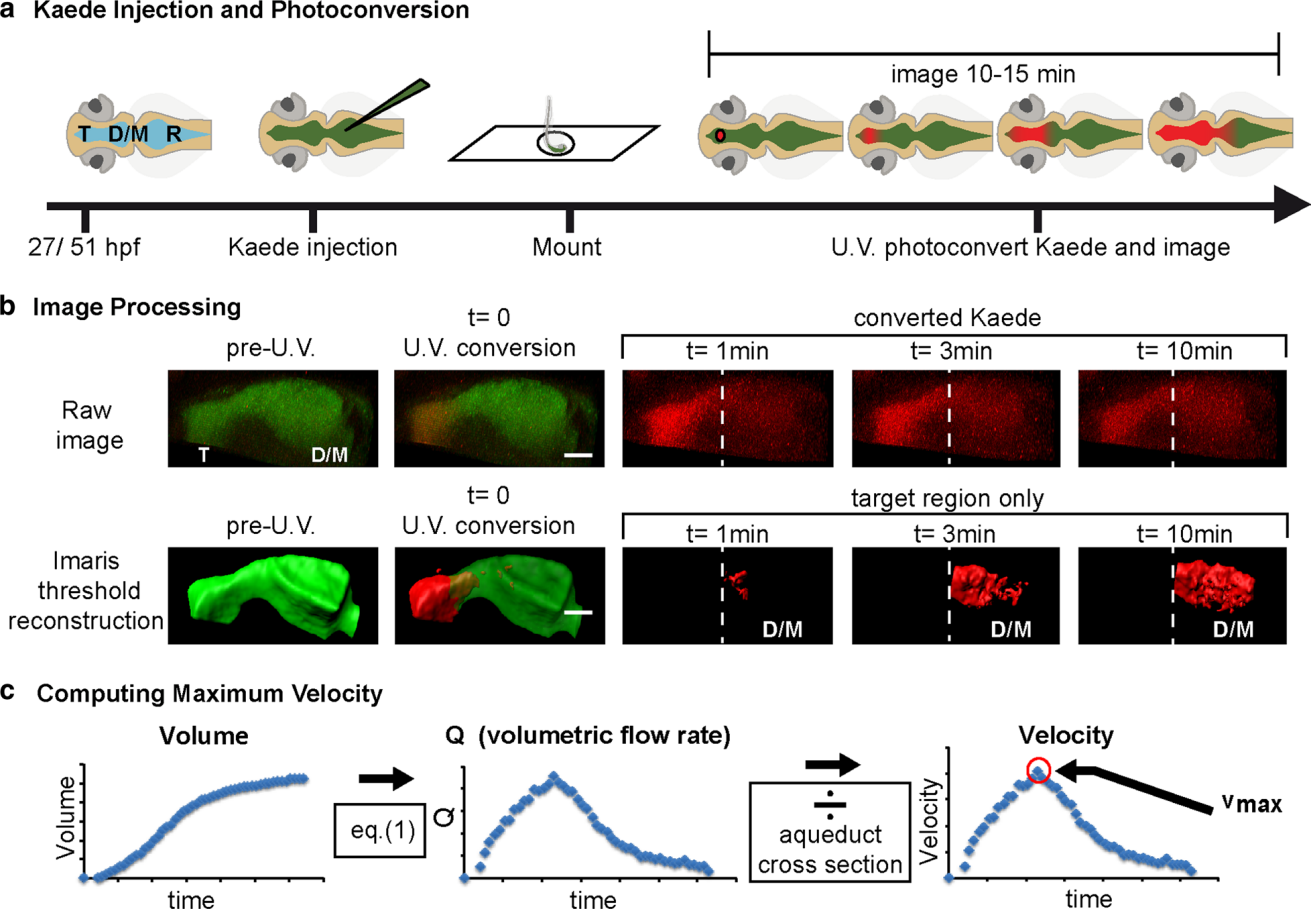
Experimental workflow. **a** Schematic representation of rhombencephalic ventricle Kaede injection and confocal photoconversion and imaging of zebrafish ventricles. **b** Workflow for image processing. Raw images were collected and then thresholded in Imaris (Bitplane) software so that red (photoconverted) Kaede was only detected after photoconversion and was not detected in the unconverted target ventricle immediately after photoconversion. Then the volume of the unconverted target ventricle was calculated over time by Imaris (Bitplane) software. *Dashed line* indicates telencephalic-to-mesencephalic/diencephalic boundary. **c** Workflow for data analysis. From volume measurements of the target ventricle, volumetric flow rate (Q) was calculated using Eq. (1) (see “Methods” section). Q was smoothed using a 9-point moving-window average. Q was then converted to linear velocity by dividing by the cross-sectional area of the smallest part of the aqueduct between the two ventricles. These CSF dynamics were reported as the maximum velocity between the two ventricles (v_max_). *T* telencephalic ventricle, *D/M* diencephalic/mesencephalic ventricle, *R* rhombencephalic ventricle, *hpf* hours post-fertilization, *V* volume, *Q* volumetric flow rate, *v* velocity. *Scale bar*: 50 μm

Larvae were anesthetized as described above and the rhombencephalic ventricle was microinjected with 2 nL of Kaede, a photoactivatable protein with fluorescence that changes from green to red upon illumination with UV light [36, 37]. After injection, larvae recovered from injection and anesthesia for at least 30 min prior to imaging, mitigating any immediate effects of dye injection. Embryos were then anesthetized as described above and placed in wells of 1 % agarose (early larva), or embedded in 1 % low-melting point agarose on a coverslip (late larva) for confocal microscopy [37]. Confocal imaging and photoconversion was performed using an inverted Zeiss LSM710 laser-scanning microscope equipped with a 25× (0.8 NA) LD LCI Plan Apochromat water-immersion objective (Fig. 1a). Kaede photoconversion was performed on a circle (diameter of 8 pixels) in the center of the ventricle of interest using the 405 nm laser at 85–95 % strength (for 700 iterations at scan speed = 7, ~20 s per stack). Stacks were collected using simultaneous activation of green and red channels and continuous acquisition for 10–15 min (Fig. 1a).

### Image processing and fluid movement quantification

Confocal time-lapse images were analyzed using Imaris software (Bitplane, Belfast, UK) to create thresholded volumetric surfaces of photoconverted Kaede. The red threshold was set for each image such that no signal was detected before photoconversion, and no signal was detected in the unconverted target ventricle at time = 0 (first scan after photoconversion) (Fig. 1b). Imaris generated volume (V) data from each time point. From these volume readings, the volumetric flow rate at time t (Q_t_) was calculated for each time point using Eq. (1):

1 $$Q_{t} = \frac{{V_{t} - V_{t - 1} }}{{T_{t} - T_{t - 1} }} = \frac{{\Delta {\text{V}}}}{\Delta T}$$

Q was then smoothed to negate technical noise using a 9-point moving window average. To control for individual variations in ventricle shape, Q_t_ was converted to linear velocity (v) by dividing Q by the cross-sectional area of the aqueduct. Cross-sectional areas were measured using Imaris software (Bitplane, Belfast, UK) by creating surfaces of the unconverted (green) Kaede signal in the first scan (before photoconversion) at the narrowest point between the two ventricles being analyzed. The maximum velocity number (v_max_) was used to analyze brain ventricle CSF movement (see Fig. 1c).

### Acute inhibition of heartbeat

Heartbeat was acutely inhibited by soaking ventricle-injected embryos in 40 mM 2,3 butanedione monoxime (BDM; Sigma, St. Louis, USA) solubilized in embryo medium [23] for 10–30 min prior to imaging as previously shown [38]. This treatment stops heartbeat, but does not change the gross ventricular anatomy within 2 h of incubation (Additional file 1: Fig. S2). BDM prevents action of all muscles (and can alter activities of connexins [39], potassium channels [40, 41], and L-type calcium channels [42]) and is therefore less precise for disruption of heartbeat than the *tnnt2a*^−*/*−^ mutant.

### Cilia imaging

The Tg(*βact:Arl13b*–GFP) transgenic line was used for visualization of cilia. Embryos were treated with 40 mM BDM, as described above for 10 min to stop heartbeat and enable completely steady, immotile embryos suitable for high-speed imaging. They were prepared for SPIM as described above and incubated in 20 mM BDM and 0.1 mg/mL Tricaine (Sigma, St. Louis, USA) in embryo medium during imaging. Images were collected at 57.22 frames per second (fps) in the telencephalon, diencephalon/mesencephalon, rhombencephalon and spinal canal. Time-lapse movies were registered using FIJI [30, 31] and the StackReg plugin [43]. The ciliary beat frequency (CBF) was calculated using the following formula: [CBF = (number of frames per second)/(average number of frames for single beat)] [44]. Images were processed by overlaying sequential frames in pseudocolor using Photoshop (Adobe).

## Results

### The early zebrafish brain ventricular system is complex and dynamic

**Fig. 2:**
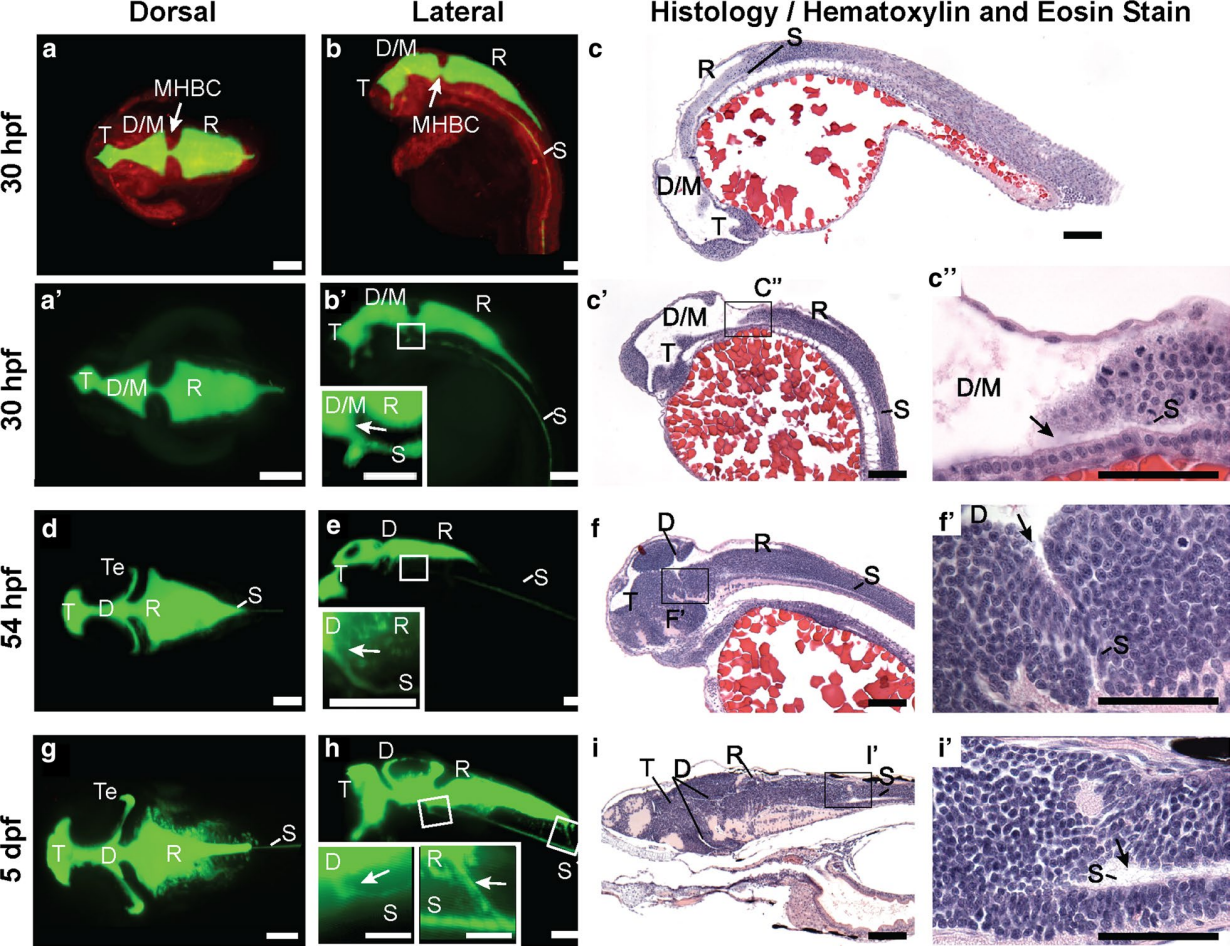
Zebrafish brain ventricle anatomy at early and late larval stages. Using 2 nL rhomencephalic ventricular injections of fluorescein-labeled dextran and imaging by Selective Plane Illumination Microscopy (SPIM, or Lightsheet Microscopy), ventricular system anatomy, volume, and connectivity was visualized in three dimensions. **a** Dorsal, **b** lateral view, 30 hpf zebrafish larvae with neuroepithelium labeled in *red* (mApple-caax) and the ventricular system labeled in *green* (fluorescein dextran injection). **c** H&E stain of 4 μm thick parasagittal section of 30 hpf zebrafish. **aʹ** Dorsal, **bʹ** Lateral view, 30 hpf larval zebrafish ventricular system. **bʹ** *inset* Magnification of *boxed* region in **bʹ** to show spinal canal (S) branching from the ventral aspect of the D/M ventricle (*arrow*). **cʹ** H&E stain of 4 μm thick section of 30 hpf zebrafish. **cʹʹ** magnification of *boxed* region in **cʹ** to show spinal canal (S) branching from D/M ventricle. **d** Dorsal, **e** Lateral view, 54 hpf larval zebrafish ventricular system. **e** *inset* Magnification of *boxed* region in **d** to show SC branching from the ventral aspect of the diencephalon (*arrow*). **f** H&E stain of 4 μm thick section of 54 hpf zebrafish. **fʹ** magnification of *boxed* region in **f** to show spinal canal (S) branching from diencephalon. **g** Dorsal, **h** Lateral view of the 5 dpf larval zebrafish ventricular system. **h** *insets* Magnification of *boxed* regions in **h** to show SC branching from the ventral aspect of the diencephalon (*arrow*) and rhombencephalon (*arrow*). **i** H&E stain of 4 μm thick section of 5 dpf zebrafish. **iʹ** magnification of *boxed* region in **i** to show spinal canal (S) branching from rhombencephalon. *T* telencephalic ventricle, *D/M* diencephalic/mesencephalic ventricle, *D* diencephalic ventricle, *Te* tectal ventricle, *R* rhombencephalic ventricle, *S* spinal canal, *hpf* hours post-fertilization, *dpf* days post-fertilization. *Scale bar*: **bʹ**, **e**, **h** *insets*, **cʹʹ**, **fʹ**, **iʹ**: 50 μm; all others: 100 μm

In order to obtain detailed structure of the zebrafish brain ventricular system, we filled the ventricles with 2000 kDa dextran conjugated to fluorescein and used SPIM to acquire images at 30 hpf (pharyngula or early larval stage; Fig. 2a, b) and 54 hpf (hatching period or late larval stage; Fig. 2d, e), prior to CP maturation but after ventricle inflation and spinal canal (SC) formation. We also examined ventricle anatomy at 5 dpf (Fig. 2g, h) after CP formation. Since the brain is still developing, the early ventricle anlagen are dynamic over these times. The telencephalic (T) ventricle is the most rostral. Early, the combined diencephalic/mesencephalic (D/M) superventricle is caudal to the T ventricle while later this middle ventricle subdivides into the diencephalic (D) and the tectal (Te) ventricles. The rhombencephalic (R) ventricle is at the most caudal aspect of the brain [22, 45] (Fig. 2). At these stages, the SC branches off from the ventral aspect of the D ventricle (Fig. 2b′, c–c′′, e, f–f′). This is the first demonstration of early connection from the diencephalon to the spinal canal in any species.

Ventricular shape changes over the first 2 days of development; with the combined diencephalic/tectal (D/Te) ventricle developing a more complex shape as development proceeds allowing the two regions to be discernible (Fig. 2d, e). The ventricular volume increases over development and the total volume of the ventricular system is ~4 nL at early larval stage, ~5.5 nL at late larval stage and ~6.5 nL at 5 dpf. The rhombencephalic ventricular volume increases from ~1.5 to ~3.5 nL from 30 to 54 hpf, but stabilizes at ~3.5 nL from 2 to 5 dpf. At the early larval stage, the isthmic (D/M-to-R) canal is significantly narrower than the anterior (T-to-D/M) canal (cross-sectional area: anterior: 1667 ± 131.3 μm^2^; n = 18 vs. isthmic: 1159 ± 61.9 μm^2^; n = 17; p = 0.0016). At the late larval stage, the isthmic (D-to-R) and anterior (T-to-D) canals are smaller than at early larval stage, but are equal to one another (cross-sectional area: anterior: 665.3 ± 118.0 μm^2^; n = 17 vs. isthmic: 716.2 ± 129.5 μm^2^; n = 21; p = 0.78). By 5 dpf, the adult connection from the R ventricle to the SC is forming (Fig. 2g–iʹ). These data add detail to the anatomical understanding of the developing ventricular system and ventricular dimensions may also play a role in influencing CSF movement.

### CSF moves from anterior to posterior in early larval zebrafish brain ventricles, but partially reverses direction at late larval stages

**Fig. 3:**
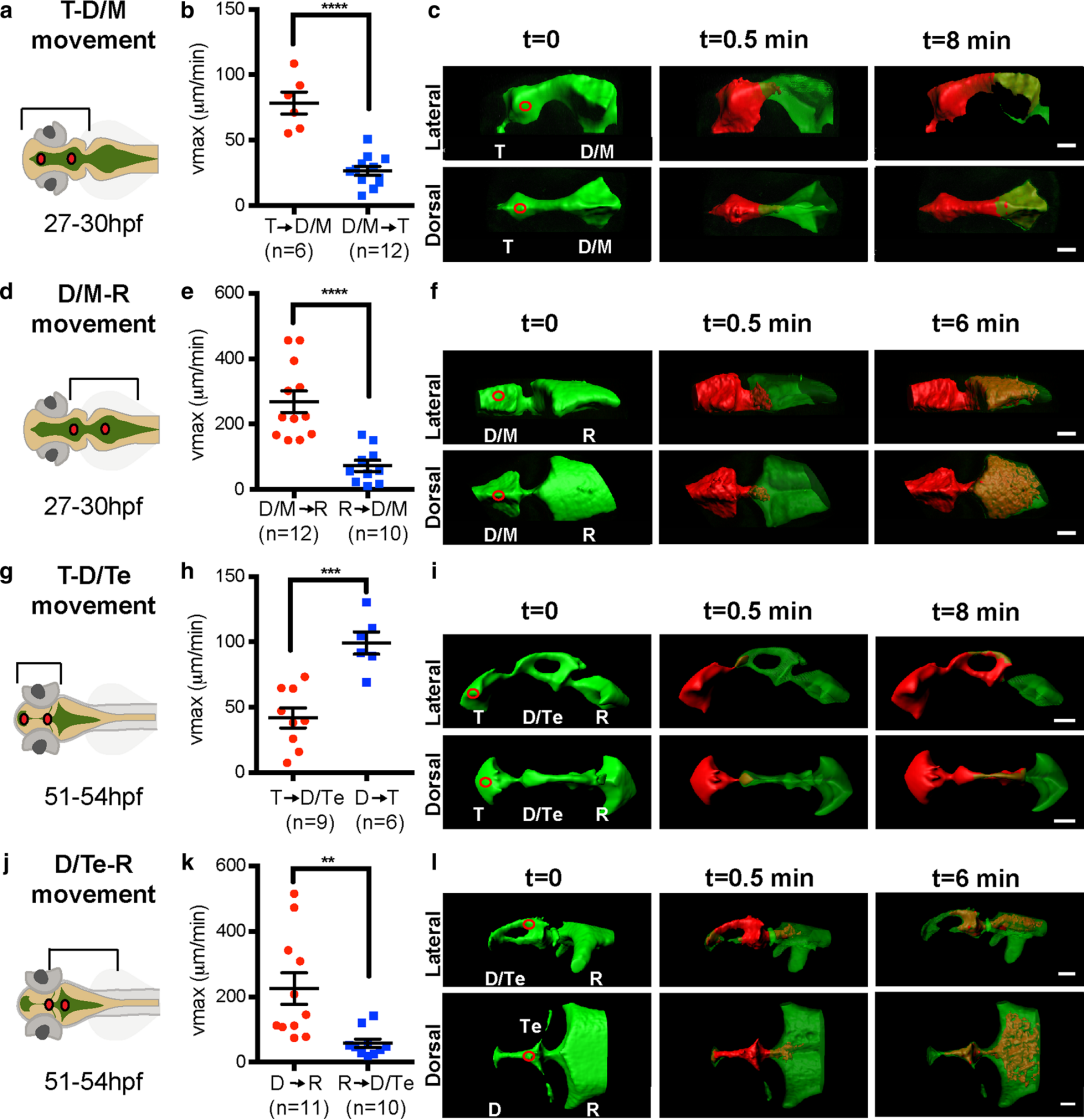
Bulk movement of CSF at early and late larval stages. **a**, **d**, **g**, **j** Schematics of brain ventricular regions analyzed. **b** At 27–30 hpf, the average v_max_ is significantly higher in the anterior to posterior (*red*) direction through the telencephalic-to-diencephalic/mesencephalic aqueduct than in the posterior to anterior direction (*blue*). **c** Overlaid thresholded volumes generated by Imaris (Bitplane) software of a representative 27–30 hpf T → D/M dataset. **e** At 27–30 hpf, the average v_max_ is significantly higher in the anterior to posterior (*red*) direction through the diencephalic/mesencephalic-to-rhombencephalic aqueduct than in the posterior to anterior direction (*blue*). **f** Overlaid thresholded volumes generated by Imaris (Bitplane) software of a representative 27–30 hpf D/M → R dataset. **h** At 51–54 hpf, through the telencephalic-to-diencephalic aqueduct, directionality is reversed such that the v_max_ is significantly higher in the posterior to anterior (*blue*) direction through the telencephalic-to-diencephalic aqueduct than in the anterior to posterior direction (*red*). **i** Overlaid thresholded volumes generated by Imaris (Bitplane) software of a representative 51–54 hpf T → D dataset. **k** At 51–54 hpf, the average v_max_ is significantly higher in the anterior to posterior (*red*) direction through the diencephalic-to-rhombencephalic aqueduct than in the posterior to anterior direction (*blue*). **l** Overlaid thresholded volumes generated by Imaris (Bitplane) software of a representative 51–54 hpf D → R dataset. *Red circles* indicate photoconversion region. *Lines* represent average v_max_ and *error bars* denote SEM. p value calculated using unpaired Student’s t test, **p < 0.001; ***p < 0.0005; ****p < 0.0001. *T* telencephalic ventricle, *D/M* diencephalic/mesencephalic ventricle, *Te* tectal ventricle, *R* rhombencephalic ventricle, *hpf* hours post-fertilization. *Scale bar*: 50 μm

To quantify CSF movement during the major period of developmental neurogenesis, we filled the ventricles with photoactivatable Kaede protein. Kaede was injected into zebrafish brain ventricles and activated by a local UV pulse at 405 nm in the T, D (D/M) or R ventricle at early or late larval stages. CSF movement was subsequently determined and v_max_ calculated (see “Methods” section, Fig. 1). At 27–30 hpf (early larva), the average v_max_ for CSF movement from T to D/M ventricle is significantly greater than for CSF movement in the reverse direction, from D/M to T ventricle (Fig. 3a–c). Preferential anterior-to-posterior movement is also observed in the D/M to R ventricle direction in early larvae (Fig. 3d–f). However, at late larval stages (51–54 hpf) the average v_max_ for CSF movement from T to D/Te ventricle is less than that of CSF movement in the D to T ventricular direction (Fig. 3g–i), indicating a reversed direction of movement between these two ventricles comparing the stages examined. The anterior-to-posterior directionality is still observed between D and R ventricles at these later stages (Fig. 3j–l).

### CSF directional movement is dependent on heartbeat at early larval stages

**Fig. 4:**
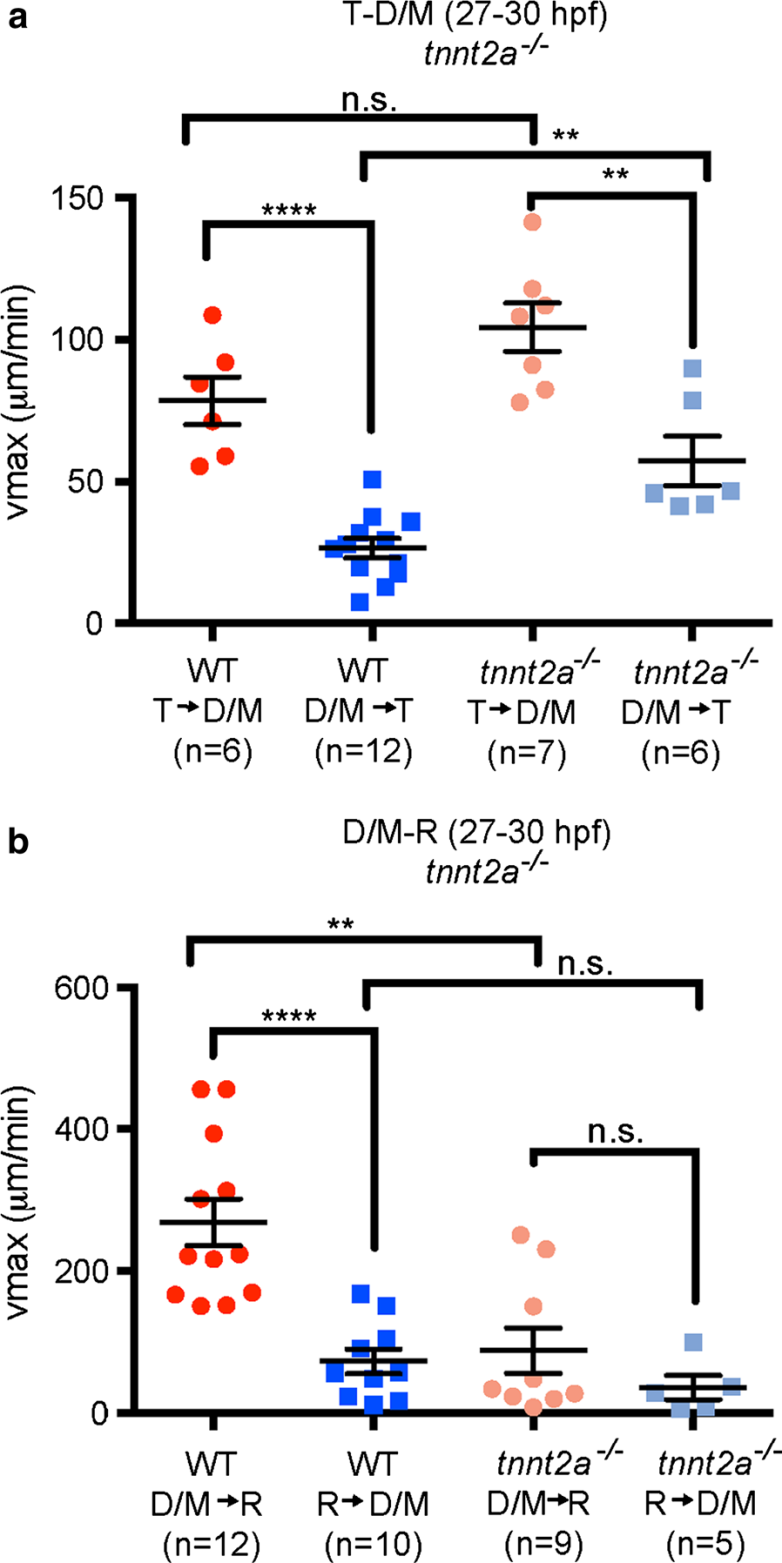
Directionality of CSF movement is partially dependent on heartbeat at early larval stage. **a** Average v_max_ for movement between the telencephalon and diencephalon/mesencephalon at 27–30 hpf in wild type (WT; *red*) and *tnnt2a* ^−*/*−^ (*pink*) from the telencephalic-to-diencephalic/mesencephalic aqueduct or the posterior to anterior direction [*blue* (WT), *light blue* (*tnnt2a* ^−*/*−^)]. **b** In *tnnt2a* ^−*/*−^ mutant fish there is no directionality of CSF movement observed through the diencephalic/mesencephalic -to-rhombencephalic aqueduct. WT data are from Fig. 3. *Horizontal lines* represent average v_max_ and *error bars* denote SEM. p value calculated using unpaired Student’s t test, **p < 0.001; ****p < 0.0001; *n.s.* not significant, *T* telencephalic ventricle, *D/M* diencephalic/mesencephalic ventricle, *R* rhombencephalic ventricle, *hpf* hours post-fertilization

Adult CSF pulses and mixes during the cardiac cycle and with breathing [15–18], zebrafish ventricle expansion and early *Xenopus* CSF movement is dependent on heartbeat [19, 34]. We therefore investigated whether directionality of CSF movement is dependent on heartbeat at early larval stages (27–30 hpf). Heartbeat was prevented genetically using the *tnnt2a*^−*/*−^ fish that never develop a heartbeat. In *tnnt2a*^−*/*−^ fish, directionality was maintained (albeit with a less significant difference), but v_max_ from D/M → T ventricle in *tnnt2a*^−*/*−^ animals is significantly higher than in WT and is unchanged from T → D/M ventricle (Fig. 4a). However, loss of *tnnt2a* function eliminated directional movement between the D/M and R ventricles and v_max_ was significantly lower from D/M → R ventricle than in WT, but unchanged from R → D/M (Fig. 4b). To acutely disrupt heartbeat, we treated WT fish with 40 mM 2,3 butanedione monoxime (BDM). In BDM-treated animals v_max_ directionality was eliminated in both T → D/M and D/M → R ventricular directions (Additional file 1: Fig. S3). BDM has a wide variety of targets that are not associated with heartbeat disruption (see “Methods” and “Discussion” sections), which might account for differences observed in CSF movement between BDM-treated and *tnnt2a*^−/−^ animals. These data suggest that the heartbeat is partially required to maintain directional CSF movement, specifically between D/M and R. In *tnnt2a*^−/−^ fish, the ventricles develop normally until 27 hpf but do not fully inflate at 36 hpf [34], suggesting brain abnormalities that could be attributed to disruption in CSF movement.

### Brain ventricle cilia are non-motile at 27 hpf and cannot contribute to directional CSF movement

**Fig. 5:**
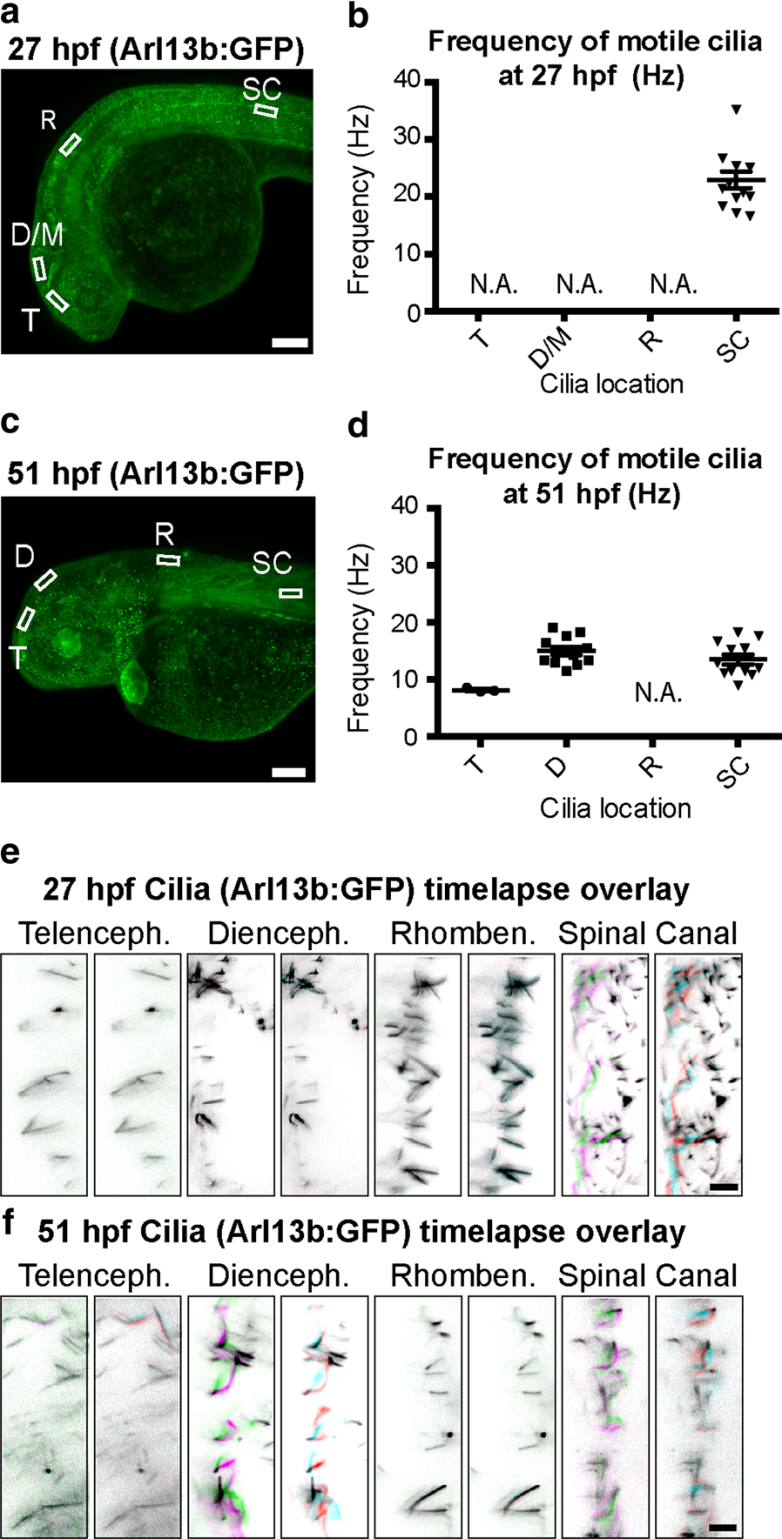
Brain cilia are non-motile at early larval stage; some are motile at late larval stage. **a**, **c** *Arl13b*: GFP is expressed in cilia throughout the developing fish including the neuroepithelium and spinal canal. **b**, **d** Quantification of ciliary beat frequency (Hz) for motile cilia in each region at early (27 hpf) and late (51 hpf) larval stages, if any. **e**, **f** Visualization of ciliary movement for cilia in each region at 27 and 51 hpf, if any. Each of the three panels is an overlay of two sequential image acquisition frames (images were taken in the same region at distinct times at 57.22 fps, so each overlay represents a time difference of ~0.017 s). See Additional file 2: Movie 1 and Additional file 3: Movie 2 for full dataset. Colors were selected such that unmoved pixels appear *black* (Frame 1: *green* + *magenta*; Frame 2: *red* + *cyan*). *Horizontal lines* represent average v_max_ and *error bars* denote SEM. *T* telencephalic ventricle, *D/M* diencephalic/mesencephalic ventricle, *R* rhombencephalic ventricle, *SC* spinal canal ventricle analysis region, *hpf* hours post-fertilization, *N.A.* quantification of cilia frequency is not applicable because no cilia are motile. *Scale bars*: **a**, **c**: 100 μm; **e**, **f**: 5 μm

It has been suggested that ciliary beating in the neuroepithelium bordering the brain ventricular lumen drives CSF movement [46]. Monociliated cells are present in the zebrafish neuroepithelium at the stages examined [25], so we investigated whether these cilia beat in a concerted fashion. Early central nervous system cilia in the zebrafish spinal canal beat at ~12 Hz [38], but motility of the cilia in the early zebrafish brain has not been characterized. Using the Tg(*βact:Arl13b*–GFP) transgenic line (Fig. 5a, c) to visualize CNS cilia, we confirmed that a subset of spinal canal cilia beat at early (27 hpf) and late (51 hpf) larval stages (Fig. 5; Additional file 2: Movie S1, Additional file 3: Movie S2). At the early larval stage, cilia in the neuroepithelium do not beat (Fig. 5b, e; Additional file 2: Movie S1). However in late larvae, motile brain cilia are observed in telencephalon and are especially active in the diencephalon (Fig. 5d, f; Additional file 3: Movie S2). We conclude that at early larval stages it is unlikely that the immotile cilia contribute to CSF movement.

## Discussion

In this study, we expanded understanding of CSF dynamics through quantification of CSF movement. Using the zebrafish model, we focused on the major period of developmental neurogenesis at two stages: early larva [pharyngula stage; 27–30 h post-fertilization (hpf)] and late larva (hatching period; 51–54 hpf). Our study leads to four conclusions: (1) CSF moves preferentially anterior to posterior from the diencephalic (D) (or combined diencephalic/mesencephalic superventricle (D/M)) to rhombencephalic (R) ventricles. (2) Direction of CSF movement between the telencephalic (T) and diencephalic (D) ventricles reverses from early to late larval stages. (3) Directional CSF movement is partially dependent on heartbeat. (4) At early larval stages no motile cilia are present in the brain and therefore are not likely to direct the CSF movement observed. Together these data indicate that locally restricted CSF factors or physical signals associated with fluid movement may contribute to specialization of the early brain.

The primarily unidirectional CSF movement observed between D (or D/M) and R ventricles was associated with a large v_max_ from D (D/M) to R ventricles. Reverse movement was present, but with about 25 % of the observed forward v_max_. This raises two questions: (1) where does the excess volume of CSF entering the R ventricle go and (2) what mechanisms underlie directional CSF movement? First, with respect to CSF “destination”, the brain-CSF barrier is quite tight at early developmental stages [47], however small molecules (<70 kDa) can pass between the ventricles and the neuroepithelium [36]. Water is also likely selectively transported since aquaporins 1a.1, 3a, 7 are expressed by the neuroepithelium [48]. Therefore some CSF components could pass in or out of the ventricular system. Additionally, we note that R ventricle volume more than doubles over this timeframe of development, perhaps due to CSF influx from more anterior ventricles as movement proceeds from D → R ventricles. Second, with respect to potential mechanisms of directional CSF movement at the stages examined, the choroid plexuses (CP) are not yet mature and there is unlikely to be active secretion from CP precursors. It is not known at what stage the CPs become mature and secrete CSF in zebrafish. Ventricles inflate at approximately 20 hpf [34] with no evidence of CPs. Henson et al. [49] claim that the zebrafish CP are mostly mature by 3 dpf, but Garcia-Lecea et al. [50] report more complete maturation and coalescing of the CP in the R ventricle by 5 dpf. However, even without secretory CP, different regions of the neuroepithelium could produce CSF at different rates to drive directional movement. Alternately, osmolarity differences between the ventricles could drive directional CSF movement, a mechanism that contributes to diffusion gradients in adult brain [51].

Directional CSF movement from D (D/M) to R ventricles could result in factors from the D (D/M) ventricle entering the R ventricle, but factors unique to the R ventricle may remain largely distinct from those of D (D/M). The stages examined encompass a major period of neurogenesis, when the detailed patterns of the diencephalon, mesencephalon, and rhombencephalon begin to read out as distinct neuronal and glial differentiation programs. Thus differentially localized CSF factors could modulate differentiation of the optic tectum (which is derived from the mesencephalic epithelium) or the cerebellum and medulla (which is derived from the rhombencephalic epithelium). At the stages examined, the spinal canal connects to the D (D/M) ventricle. We observed no converted Kaede protein in the spinal canal (data not shown), indicating that the vast majority of D (D/M) CSF movement is into the R ventricle, consistent with the smaller diameter of the spinal canal connector relative to the isthmic canal.

A surprising contrast to the consistent direction of movement between D (D/M) and R ventricles was the reversal of direction between T and D (D/M) ventricles when early and late larval stages were examined. What would lead to this reversal? One possibility is that at late larval stages, CP cells present along the D ventricle, which arise later but complete differentiation first, have begun to coalesce and are producing CSF, while the CP cells in the R and T ventricles have not yet completed differentiation [50, 52]. This could lead to greater pressure from in the D ventricle from more mature CP cells and promote CSF entry into the T ventricle, where the CP is the last to form [31]. Therefore, posterior-to-anterior CSF movement between D and T ventricles could result from active CSF secretion and greater D ventricular CSF pressure. The strong ciliary movement in D neuroepithelium relative to T neuroepithelium at late larva stage may also contribute to reversal of flow. Another possibility is that the reverse flow could result from gradually increasing pressure in the D ventricle as result of ventricular shape change and the subsequent reduction in D ventricular volume as development proceeds. Whatever the mechanism, the outcome of this change in direction would be that at early larval stages CSF in the D ventricle would contain factors derived from the T, whereas at late larval stages CSF in the T ventricle would contain some components derived from the D. Thus distinct cohorts of factors in T or D ventricles could be present at each stage.

Heartbeat plays a role in CSF dynamics early in brain development, as has also been shown in *Xenopus* [19]. After *tnnt2a* loss of function, a mechanism independent of heartbeat, perhaps CSF production, maintains directional CSF movement from T and D/M ventricles, but not for D/M to R ventricular movement, although there is a trend toward less significant directionality. Consistently, acute loss of heartbeat with BDM treatment disrupts all directional CSF movement. However, BDM prevents action of all muscles (and can alter activities of connexins [39], potassium channels [40, 41] and l-type calcium channels [42]) and some of these side effects may contribute to the phenotypes observed.

At early larval stages no motile cilia are present in the neuroepithelium and are therefore unlikely to contribute to the CSF movement observed. The non-motile cilia at this time may be able to detect CSF movement, potentially to activate planar cell polarity signaling described by Ohata and colleagues [21], or to guide migrating neurons or extending axons [54]. At late larval stages, cilia along the D ventricle are highly motile and may indicate differentiating CP, as CP cells have motile cilia [52]. As discussed above, these ciliary movements or CSF production may drive CSF movement from D to T ventricles. Mutants that disrupt the primary cilia present with hydrocephalus [53], which would preclude clear comparison of CSF movement. However, future studies acutely disrupting cilia are needed to fully address potential roles of cilia in CSF movement.

In summary, this study demonstrates directional CSF movement during early brain development. One outcome of this may be differential concentrations of CSF factors in different ventricles, which may contribute to regional specialization of the brain. Directional CSF movement may also act as a physical signal to modulate neuroepithelial fates. The zebrafish offers an excellent system with which to address these intriguing possibilities.

## Conclusion

Embryonic CSF in the zebrafish model moves directionally during the major period of developmental neurogenesis. Specifically, CSF moves preferentially from the diencephalic into the rhombencephalic ventricle. In addition, direction of CSF movement between telencephalic and diencephalic ventricles reverses from early [pharyngula stage; 27–30 h post-fertilization (hpf)] to late (hatching period; 51–54 hpf) larval stages. CSF movement is partially dependent on heartbeat. At early larval stages, the absence of motile cilia indicates that cilia likely do not direct CSF movement. These data suggest that CSF components may be compartmentalized and could contribute to specialization of the early brain and CSF movement may also provide directional mechanical cues.

## Additional files

10.1186/s12987-016-0036-z Ventricle injections do not disrupt gross ventricular morphology; **Fig. S2.** BDM treatment does not disrupt gross ventricular morphology over 2 hours; **Fig. S3.** BDM treatment disrupts CSF directional movement at early larval stage.
10.1186/s12987-016-0036-z Cilia movement at 27 hpf.
10.1186/s12987-016-0036-z Cilia movement at 51 hpf.

## Abbreviations

BDM: 2,3 butanedione monoxime
CP: choroid plexus
CSF: cerebrospinal fluid
D: diencephalon
D/M: combined diencephalic/mesencephalic superventricle
dpf: days post-fertilization
hpf: hours post-fertilization
Q: volumetric flow rate
R: rhombencephalon
SC: spinal canal
SPIM: Selective Plane Illumination Microscopy
T: telencephalon
v: velocity
V: volume
WT: wild-type animals

## References

[1] Martín C, Bueno D, Alonso MI, Ja Moro, Callejo S, Parada C (2006). FGF2 plays a key role in embryonic cerebrospinal fluid trophic properties over chick embryo neuroepithelial stem cells. Dev Biol.

[2] Huang X, Liu J, Ketova T, Fleming JT, Grover VK, Cooper MK (2010). Transventricular delivery of Sonic hedgehog is essential to cerebellar ventricular zone development. Proc Natl Acad Sci USA.

[3] Lehtinen MK, Zappaterra MW, Chen X, Yang YJ, Hill AD, Lun M (2011). The cerebrospinal fluid provides a proliferative niche for neural progenitor cells. Neuron.

[4] Parada C, Gato A, Bueno D (2008). All-trans retinol and retinol-binding protein from embryonic cerebrospinal fluid exhibit dynamic behaviour during early central nervous system development. NeuroReport.

[5] Chau KF, Springel MW, Broadbelt KG, Park HY, Topal S, Lun MP (2015). Progressive differentiation and instructive capacities of amniotic fluid and cerebrospinal fluid proteomes following neural tube closure. Dev Cell.

[6] Zappaterra MD, Lisgo SN, Lindsay S, Gygi SP, Walsh CA, Ballif BA (2007). A comparative proteomic analysis of human and rat embryonic cerebrospinal fluid. J Proteome Res.

[7] Chang JT, Lehtinen MK, Sive H (2016). Zebrafish cerebrospinal fluid mediates cell survival through a retinoid signaling pathway. Dev Neurobiol.

[8] Feliciano DM, Zhang S, Nasrallah CM, Lisgo SN, Bordey A (2014). Embryonic cerebrospinal fluid nanovesicles carry evolutionarily conserved molecules and promote neural stem cell amplification. PLoS One.

[9] Gato Á, Moro JA, Alonso MI, Bueno D, De La Mano A, Martín C (2005). Embryonic cerebrospinal fluid regulates neuroepithelial survival, proliferation, and neurogenesis in chick embryos. Anat Rec A Discov Mol Cell Evol Biol.

[10] O’Rahilly R, Muller F (1990). Ventricular system and choroid plexuses of the human brain during the embryonic period proper. Am J Anat.

[11] Brinker T, Stopa E, Morrison J, Klinge P (2014). A new look at cerebrospinal fluid circulation. Fluids and Barriers CNS.

[12] Pollay M, Curl F (1967). Secretion of cerebrospinal fluid by the ventricular ependyma of the rabbit. Am J Physiol.

[13] Sonnenberg H, Solomon S, Frazier DT (1967). Sodium and chloride movement into the central canal of cat spinal cord. Proc Soc Exp Biol Med.

[14] Davson H (1967). The physiology of the cerebrospinal fluid.

[15] Bakshi R, Caruthers SD, Janardhan V, Wasay M (2000). Intraventricular CSF pulsation artifact on fast fluid-attenuated inversion-recovery MR images: analysis of 100 consecutive normal studies. AJNR Am J Neuroradiol.

[16] Bradley WG, Scalzo D, Queralt J, Nitz WN, Atkinson DJ, Wong P (1996). Normal-pressure hydrocephalus: evaluation with cerebrospinal fluid flow measurements at MR imaging. Radiology.

[17] Sherman JL, Citrin CM, Gangarosa RE, Bowen BJ (1987). The MR appearance of CSF flow in patients with ventriculomegaly. AJR Am J Roentgenol.

[18] Brinker T, Ludemann W, Berens von Rautenfeld D, Samii M (1997). Dynamic properties of lymphatic pathways for the absorption of cerebrospinal fluid. Acta Neuropathol.

[19] Miskevich F (2010). Imaging fluid flow and cilia beating pattern in Xenopus brain ventricles. J Neurosci Methods.

[20] Hagenlocher C, Walentek P, Ller C, Thumberger T, Feistel K (2013). Ciliogenesis and cerebrospinal fluid flow in the developing Xenopus brain are regulated by foxj1. Cilia.

[21] Ohata S, Herranz-Perez V, Nakatani J, Boletta A, Garcia-Verdugo JM, Alvarez-Buylla A (2015). Mechanosensory genes Pkd1 and Pkd2 contribute to the planar polarization of brain ventricular epithelium. J Neurosci.

[22] Turner MH, Ullmann JF, Kay AR (2012). A method for detecting molecular transport within the cerebral ventricles of live zebrafish (*Danio rerio*) larvae. J Physiol.

[23] Westerfield M (1995). The zebrafish book. a guide for the laboratory use of zebrafish (*Danio rerio*).

[24] Kimmel CB, Ballard WW, Kimmel SR, Ullmann B, Schilling TF (1995). Stages of embryonic development of the zebrafish. Dev Dyn.

[25] Borovina A, Superina S, Voskas D, Ciruna B (2010). Vangl2 directs the posterior tilting and asymmetric localization of motile primary cilia. Nat Cell Biol.

[26] Clark KJ, Balciunas D, Pogoda HM, Ding Y, Westcot SE, Bedell VM (2011). In vivo protein trapping produces a functional expression codex of the vertebrate proteome. Nat Methods.

[27] Muntean BS, Horvat CM, Behler JH, Aboualaiwi WA, Nauli AM, Williams FE (2010). A comparative study of embedded and anesthetized zebrafish in vivo on myocardiac calcium oscillation and heart muscle contraction. Front Pharmacol.

[28] Strykowski JL, Schech JM (2015). Effectiveness of recommended euthanasia methods in larval zebrafish (Danio rerio). J Am Assoc Lab Anim Sci.

[29] Denvir MA, Tucker CS, Mullins JJ (2008). Systolic and diastolic ventricular function in zebrafish embryos: influence of norepenephrine, MS-222 and temperature. BMC Biotechnol.

[30] Schneider CA, Rasband WS, Eliceiri KW (2012). NIH Image to ImageJ: 25 years of image analysis. Nat Methods.

[31] Schindelin J, Arganda-Carreras I, Frise E, Kaynig V, Longair M, Pietzsch T (2012). Fiji: an open-source platform for biological-image analysis. Nat Methods.

[32] Preibisch S, Amat F, Stamataki E, Sarov M, Singer RH, Myers E (2014). Efficient Bayesian-based multiview deconvolution. Nat Methods.

[33] Preibisch S, Saalfeld S, Schindelin J, Tomancak P (2010). Software for bead-based registration of selective plane illumination microscopy data. Nat Methods.

[34] Lowery LA, Sive H (2005). Initial formation of zebrafish brain ventricles occurs independently of circulation and requires the nagie oko and snakehead/atp1a1a.1 gene products. Development..

[35] Sheehan DC, Hrapchak BB (1980). Theory and practice of histotechnology.

[36] Chang JT, Sive H (2012). An assay for permeability of the zebrafish embryonic neuroepithelium. J Vis Exp.

[37] Gutzman JH, Sive H (2009). Zebrafish brain ventricle injection. J Vis Exp.

[38] Kramer-Zucker AG, Olale F, Haycraft CJ, Yoder BK, Schier AF, Drummond IA (2005). Cilia-driven fluid flow in the zebrafish pronephros, brain and Kupffer’s vesicle is required for normal organogenesis. Development.

[39] Verrecchia F, Herve JC (1997). Reversible blockade of gap junctional communication by 2,3-butanedione monoxime in rat cardiac myocytes. Am J Physiol.

[40] Schlichter LC, Pahapill PA, Chung I (1992). Dual action of 2,3-butanedione monoxime (BDM) on K+ current in human T lymphocytes. J Pharmacol Exp Ther.

[41] Lopatin AN, Nichols CG (1993). 2,3-Butanedione monoxime (BDM) inhibition of delayed rectifier DRK1 (Kv2.1) potassium channels expressed in Xenopus oocytes. J Pharmacol Exp Ther.

[42] Ferreira G, Artigas P, Pizarro G, Brum G (1997). Butanedione monoxime promotes voltage-dependent inactivation of l-type calcium channels in heart. Effects on gating currents. J Mol Cell Cardiol.

[43] Thevenaz P, Ruttimann UE, Unser M (1998). A pyramid approach to subpixel registration based on intensity. IEEE Trans Image Process.

[44] Chilvers MA, O’Callaghan C (2000). Analysis of ciliary beat pattern and beat frequency using digital high speed imaging: comparison with the photomultiplier and photodiode methods. Thorax.

[45] Butler AB, Hodos W (2005). Comparative vertebrate neuroanatomy: evolution and adaptation.

[46] Narita K, Takeda S (2015). Cilia in the choroid plexus: their roles in hydrocephalus and beyond. Front Cell Neurosci.

[47] Stolp HB, Liddelow SA, Sa-Pereira I, Dziegielewska KM, Saunders NR (2013). Immune responses at brain barriers and implications for brain development and neurological function in later life. Front Integr Neurosci.

[48] Thisse B, Heyer V, Lux A, Alunni V, Degrave A, Seiliez I (2004). Spatial and temporal expression of the zebrafish genome by large-scale in situ hybridization screening. Methods Cell Biol.

[49] Henson HE, Parupalli C, Ju B, Taylor MR (2014). Functional and genetic analysis of choroid plexus development in zebrafish. Front Neurosci.

[50] Garcia-Lecea M, Kondrychyn I, Fong SH, Ye ZR, Korzh V (2008). In vivo analysis of choroid plexus morphogenesis in zebrafish. PLoS One.

[51] Bito LZ, Davson H (1966). Local variations in cerebrospinal fluid composition and its relationship to the composition of the extracellular fluid of the cortex. Exp Neurol.

[52] Lun MP, Monuki ES, Lehtinen MK (2015). Development and functions of the choroid plexus-cerebrospinal fluid system. Nat Rev Neurosci.

[53] Choksi SP, Babu D, Lau D, Yu X, Roy S (2014). Systematic discovery of novel ciliary genes through functional genomics in the zebrafish. Development.

[54] Sawamoto K, Wichterle H, Gonzalez-Perez O, Cholfin JA, Yamada M, Spassky N (2006). New neurons follow the flow of cerebrospinal fluid in the adult brain. Science..

