# Supplementary material for: Directional cerebrospinal fluid movement between brain ventricles in larval zebrafish
Source: Fluids Barriers CNS. 2016 Jun 21;13:11. doi: 10.1186/s12987-016-0036-z (PMC4915066; doi:10.1186/s12987-016-0036-z)
Supplement: Supplementary file 1 — 10.1186/s12987-016-0036-z Ventricle injections do not disrupt gross ventricular morphology; Fig. S2. BDM treatment does not disrupt gross ventricular morphology over 2 hours; Fig. S3. BDM treatment disrupts CSF directional movement at early larval stage. [file 12987_2016_36_MOESM1_ESM.pdf]

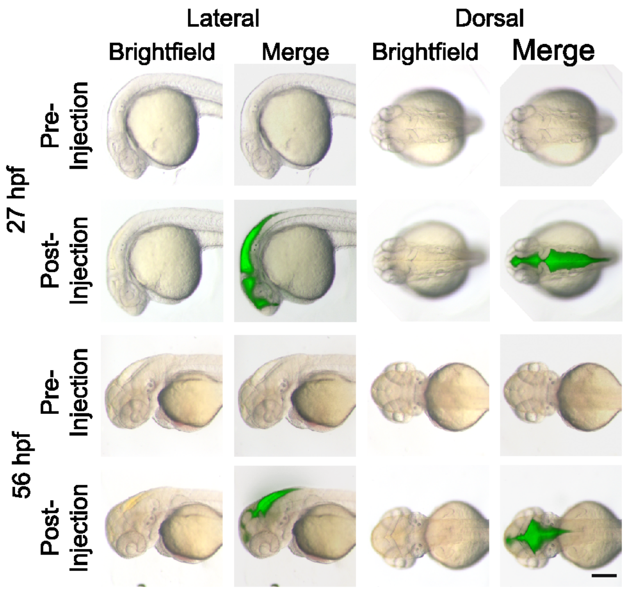

Figure S1: Ventricle injections do not disrupt gross ventricular morphology

Larvae were imaged under brightfield (first and third columns) and fluorescence (second and fourth columns, merged with brightfield) microscopy both before and after ventricular injection. Injections comprised 2nL of 2000kDa dextran FITC at 27 hpf (top) and 56 hpf (bottom). No change in ventricular morphology is observed in either dorsal (first two columns) or lateral (last two columns) views. hpf, hours post-fertilization. Scale bar: 200μm. N ≥10 observed at each stage, representative images shown.

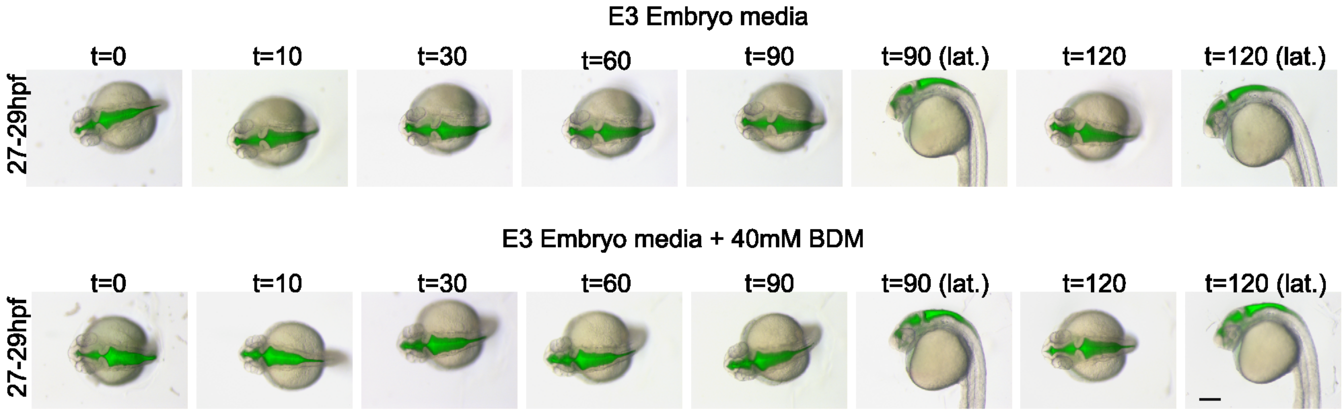

<u>Figure S2: BDM treatment does not disrupt gross ventricular morphology over 2 hours</u>

Larvae treated with 40mM BDM show no change in gross ventricular shape compared to untreated larvae. Brightfield and florescence microscopy (merged images shown) is shown after ventricular injection of 2nL of 2000kDa dextran FITC. BDM, 2,3 butanedione monoxime ; hpf, hours post-fertilization; t= time after treatment (minutes). Scale bar: 200μm. N ≥10 observed for each treatment, representative images shown.

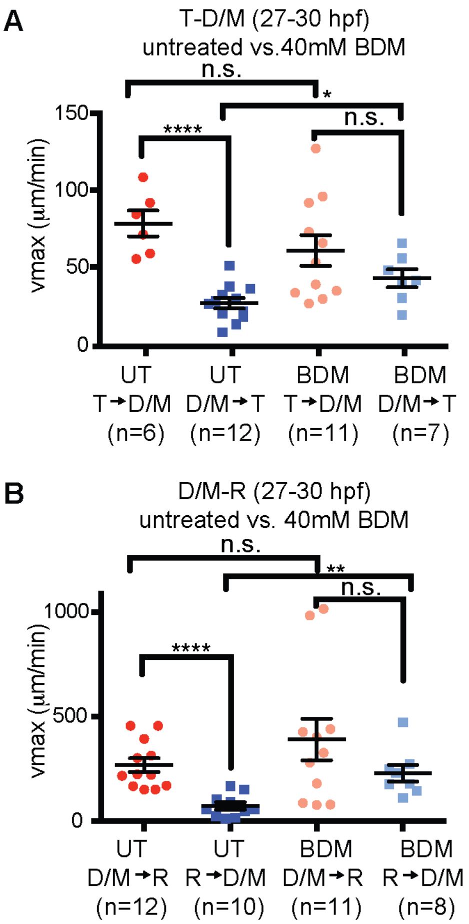

<u>Figure S3: BDM treatment disrupts CSF directional movement at early larval stage</u>

(A) At 27-30 hpf in BDM-treated fish, no directional CSF movement is observed through either the telencephalic-to-diencephalic/mesencephalic (D/M → T and T→ D/M) aqueduct nor (B) through the diencephalic/mesencephalic -to-rhombencephalic aqueduct (D/M → R and R→ D/M)) red/blue (WT), pink/light blue (BDM-treated)). Horizontal lines represent average v_{max} and error bars denote SEM. p-value calculated using unpaired Student's t test, ** $p < 0.001$. BDM, 2,3-butanedione monoxime; T, telencephalic ventricle; D/M, diencephalic/ mesencephalic ventricle; R, rhombencephalic ventricle; hpf, hours post-fertilization.
